# A Comparative Study of Drosophila and Human A-Type Lamins

**DOI:** 10.1371/journal.pone.0007564

**Published:** 2009-10-26

**Authors:** Sandra R. Schulze, Beatrice Curio-Penny, Sean Speese, George Dialynas, Diane E. Cryderman, Caitrin W. McDonough, Demet Nalbant, Melissa Petersen, Vivian Budnik, Pamela K. Geyer, Lori L. Wallrath

**Affiliations:** 1 Department of Biology, Western Washington University, Bellingham, Washington, United States of America; 2 Department of Biochemistry, University of Iowa, Iowa City, Iowa, United States of America; 3 Department of Neurobiology, University of Massachusetts, Wochester, Massachusetts, United States of America; Duke University, United States of America

## Abstract

Nuclear intermediate filament proteins, called lamins, form a meshwork that lines the inner surface of the nuclear envelope. Lamins contain three domains: an N-terminal head, a central rod and a C-terminal tail domain possessing an Ig-fold structural motif. Lamins are classified as either A- or B-type based on structure and expression pattern. The Drosophila genome possesses two genes encoding lamins, *Lamin C* and *lamin Dm_0_*, which have been designated A- and B-type, respectively, based on their expression profile and structural features. In humans, mutations in the gene encoding A-type lamins are associated with a spectrum of predominantly tissue-specific diseases known as laminopathies. Linking the disease phenotypes to cellular functions of lamins has been a major challenge. Drosophila is being used as a model system to identify the roles of lamins in development. Towards this end, we performed a comparative study of Drosophila and human A-type lamins. Analysis of transgenic flies showed that human lamins localize predictably within the Drosophila nucleus. Consistent with this finding, yeast two-hybrid data demonstrated conservation of partner-protein interactions. Drosophila lacking A-type lamin show nuclear envelope defects similar to those observed with human laminopathies. Expression of mutant forms of the A-type Drosophila lamin modeled after human disease-causing amino acid substitutions revealed an essential role for the N-terminal head and the Ig-fold in larval muscle tissue. This tissue-restricted sensitivity suggests a conserved role for lamins in muscle biology. In conclusion, we show that (1) localization of A-type lamins and protein-partner interactions are conserved between Drosophila and humans, (2) loss of the Drosophila A-type lamin causes nuclear defects and (3) muscle tissue is sensitive to the expression of mutant forms of A-type lamin modeled after those causing disease in humans. These studies provide new insights on the role of lamins in nuclear biology and support Drosophila as a model for studies of human laminopathies involving muscle dysfunction.

## Introduction

Lamins are type V intermediate filament proteins that line the inner surface of the nuclear envelope of animal cells, providing structural support and making contacts with chromatin [1], [2]. Humans possess two types of lamins, A- and B-types, which differ in developmental expression patterns and structural properties. Human A-type lamins include Lamin A and Lamin C, alternatively spliced products from the *LMNA* gene. Human B-type lamins include lamin B1 and Lamin B2, encoded by two separate genes. With the exception of Lamin C, human lamins possess a CaaX box (C, cysteine; a, aliphatic amino acid; X, any amino acid) that becomes prenylated and provides a nuclear envelope anchor. For Lamin A, the prenylated C-terminus is removed by processing by the conserved metalloprotease FACE1 (mouse Zmpste24) [3]. Thus, processed A-type lamins lack a membrane anchor that is possessed by B-type lamins.

A- and B-type lamins exhibit contrasting developmental expression profiles. B-type lamins are ubiquitously expressed throughout development. In many organisms, expression of A-type lamins does not appear until midway through embryogenesis, suggesting a role in differentiation [4], [5]. Another distinction between A- and B-type lamins has been observed by RNAi knock-down in cultured cells [6]. Depletion of both B-type lamins leads to cell death. In contrast, knock-down of A-type lamins has no effect on cell viability (Harborth et al 2001). Taken together, these data suggest that B-type lamins play an essential role, while A-type lamins have specialized functions in differentiated tissues.

Lamins possess a conserved structural organization consisting of a small N-terminal head domain, a long central alpha-helical rod domain, and a large C-terminal tail containing an immunoglobulin (Ig)-fold [7]. The Ig-fold is a conserved structural motif that interacts with nuclear proteins and DNA [7], [8]. Lamins form dimers along their rod domains, which organize into higher-order orthogonal assemblies via unknown mechanisms to generate a meshwork known as the lamina [9].

Lamins interact with a diverse set of proteins including histones, transcription factors and proteins embedded within the nuclear envelope such as those possessing LEM (Lap2, Emerin and MAN1) domains [10]. The LEM domain is an approximately 40 amino acid sequence motif that interacts with the small double stranded DNA binding protein Barrier-to-Autointegration Factor (BAF) [11], [12], [13]. Proper localization of the LEM domain protein emerin is dependent on A-type lamins [14].

Mutations in several genes encoding nuclear envelope proteins cause diseases known as laminopathies, many of which are tissue-specific. Paradoxically, these genes are nearly ubiquitously expressed [15]. Many of these diseases arise due to mutations in *LMNA*, such as autosomal dominant Emery-Dreifuss muscular dystrophy (AD-EDMD) and familial dilated cardiomyopathy (DCM), which predominantly affect muscle [16]. This observation led to the hypothesis that mutations in the lamina weaken nuclear structure, which may manifest as a dystrophic phenotype in tissues subject to mechanical stress, such as skeletal or cardiac muscle [17]. In addition to physical weakening, force-related changes in gene expression have also been observed [18]. However, susceptibility to mechanical stress cannot explain laminopathies that do not affect muscle, such as those that cause familial partial lipodystrophy (FPLD), the neuropathy Charcot Marie-tooth syndrome type 2B (CMT) and the premature aging disorder Hutchinson Gilford-Progeria Syndrome (HGPS) [16]. With the exception of HGPS, many of these laminopathies are tissue-specific, an observation that supports the hypothesis that lamins regulate tissue-specific gene expression programs, presumably by organizing chromatin and/or interacting with transcriptional regulators [17].

All vertebrates characterized to date possess A- and B-type lamins. Evolutionary analyses indicate that with the exception of Drosophila, invertebrates generally possess a single B-type lamin possessing a CaaX box [19], [20]. The *Drosophila melanogaster* genome encodes two lamin genes. *Lamin Dm_0_* encodes a protein classified as a B-type lamin based on possession of a CaaX box and ubiquitous expression throughout development [21]. *Lamin C* encodes a protein classified as an A-type lamin based on the lack of a CaaX box and developmentally regulated expression that first appears late in embryogenesis [21]. From an evolutionary perspective, A-type lamins are thought to have arisen independently twice during evolution, once in the arthropod and once in the vertebrate lineages. This hypothesis suggests that Drosophila *Lamin C* is most closely related to Drosophila *lamin Dm_0_*, which is presumably derived from an ancestral B-type lamin [22]. However the similarities between both Drosophila *Lamin C* and human *LMNA* are striking. The Drosophila *Lamin C* gene possesses shared intron/exon positions with vertebrate *LMNA*, whereas Drosophila *lamin Dm_0_* possesses totally unique intron positions [19]. Drosophila is the only known example of an invertebrate metazoan that evolved a requirement for two lamin types. Based on expression, lamin Dm_0_ appears to have a more general role whereas Lamin C possesses tissue-specific roles during differentiation. The fact that Lamin C lacks a CaaX box and localizes to the nuclear interior, in addition to the periphery, suggests the possibility of broader roles in nuclear processes.

The function of Drosophila lamins has been assessed through the analysis of mutant alleles. Homozygotes for loss of function alleles of lamin Dm_0_ exhibit a late pupal lethal phase, with a fraction of the progeny surviving to adulthood [23]. The pupal lethal phase is likely due to the fact that lamin Dm_o_ is maternally supplied [21]. “Escaper” adults display sterility, shortened lifespan, and locomotion defects [24]. At the cellular level, *lamin Dm_0_* mutants display clustering of nuclear pores, fragmented nuclear envelopes and annulate lamellae [22], [24]. There is additional evidence that lamin Dm_0_ plays a role in nuclear positioning in developing the oocyte and eye [25], [26]. It is not known whether physical abnormalities of the nuclear envelope cause these phenotypes or whether the defects are due to indirect effects of altered tissue-specific gene expression. A role for lamins in chromosome organization and gene regulation is suggested by DamID experiments in which a *Dam* methyltransferase-lamin Dm_0_ fusion protein associated with clusters of repressed genes [27].

Less is known about the phenotype of flies lacking *Lamin C*. Using imprecise P-element excision, we generated null alleles of *Lamin C* [28]. Homozygous nulls exhibit lethality during the larval to pupal stages. These findings are similar to those in mice where *lmna^−/−^* mice were shown to be lethal [29]. Here, we further characterize the *Lamin C* mutant.

We have initiated studies in Drosophila to examine the tissue-specific roles of A-type lamin with implications for understanding human disease etiology. Drosophila is a genetically tractable, well-characterized model that possesses A- and B-type lamins that have approximately 30% amino acid sequence identity with human lamins. In addition, other components of the nuclear envelope such as LEM domain proteins and BAF are conserved between humans and Drosophila [30], [31], [32], [33]. We examine the evolutionary conservation of human and Drosophila nuclear envelope proteins by expressing human lamins and the LEM domain protein emerin in Drosophila. The mammalian proteins show predictable patterns of localization suggesting shared assembly mechanisms. Supporting this observation, yeast two-hybrid analyses confirmed that human and Drosophila nuclear envelope proteins possess conserved partner interactions. We exploit similarities between arthropod and mammalian lamins to investigate the role of A-type lamins in development. Loss of Drosophila Lamin C causes defects in nuclear structure and lethality at the larval to pre-pupal stage. To model laminopathies, transgenic flies were generated that express mutant forms of Lamin C analogous to those causing disease in humans. Expression of mutant forms of Lamin C in larval muscle, but not other tissues, caused lethality. The requirement for Drosophila A-type lamin in muscle suggests conserved functions in muscle biology.

## Results

### Conserved interactions between Drosophila and human nuclear envelope proteins

**Figure 1:**
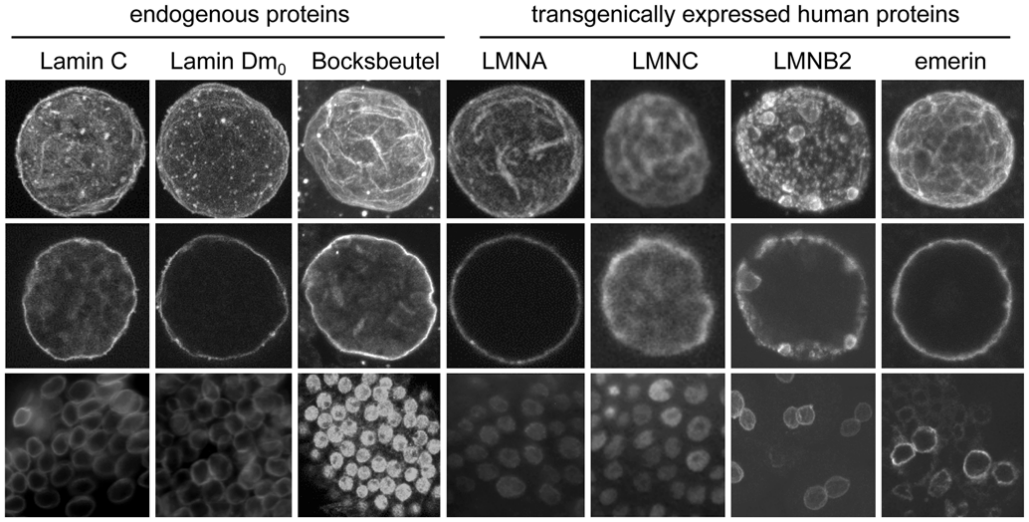
Localization of human nuclear envelope proteins in Drosophila. Polytene nuclei from salivary glands (top row z-series; middle row section) and diploid cells from imaginal discs (bottom row) were obtained from transgenic stocks and stained with antibodies specific for the human nuclear envelope proteins. All of the human proteins localized to the Drosophila nuclear envelope, with Lamin B2 showing aggregation.

Towards the development of an invertebrate model for laminopathies, we investigated the conserved relationship between Drosophila and human nuclear envelope proteins. Transgenic flies were generated that possess a single copy transgene encoding a human nuclear envelope protein under control of a heat shock promoter (*hsp70*). Multiple transgenic stocks expressing each human nuclear envelope protein were tested to rule out effects due to the site of transgene insertion. Daily heat shock treatment resulted in stable production of the human proteins and viable adults. Cytological studies were performed on salivary gland nuclei to compare the localization of the human proteins to their Drosophila counterparts (Fig. 1). Human Lamin A exhibited strict localization to the nuclear periphery in both polytene and diploid cells of transgenic third instar larvae. In contrast, Lamin C showed both peripheral and internal localization, similar to endogenous Drosophila Lamin C, suggesting that internal localization results from the absence of a CaaX box. While Lamin B2 localized to the periphery, small nuclear aggregates were also observed. [Human Lamin B1 transgenics were not analyzed by cytology due to the fact that we were unable to identify an antibody that specifically recognized Lamin B1 following expression in Drosophila.]

We expressed the LEM domain protein emerin in Drosophila, as mutations in emerin cause EDMD [34]. Heat shock induced expression of emerin resulted in localization to the nuclear periphery without visible phenotypes or lethality (Fig. 1). Thus, the mechanisms for localizing this LEM domain-containing protein appeared to be conserved. These data suggest that transgenic Drosophila might be useful as an *in vivo* model to analyze the function of mutant forms of emerin and examining the function of emerin in different mutant backgrounds.

The human nuclear envelope proteins were also expressed using the Gal4-UAS system [35]. This system involves crossing Gal4 “driver stocks” that express the Gal4 transcription factor in specific patterns to “responder stocks” possessing a transgene driven by a UAS element. The UAS element is bound by Gal4, which results in activation of transgene expression. Resulting progeny are assayed for effects of expression of the responder gene in specific patterns. UAS responder transgenes were constructed from Lamin A, C, B1, B2 and emerin, and crossed to a variety of ubiquitous and tissue-specific drivers (Table S1). Using this system, the human proteins showed nuclear localization patterns consistent with that obtained following heat shock induction. In contrast to the viability observed with the heat shock driven expression, ubiquitous and muscle-specific drivers caused lethality, primarily when used to express human B-type lamin (Table S1). Differences in the outcome between the heat shock and the Gal4-driven expression is likely due to the higher levels of protein produced by Gal4 as observed by western analysis (data not shown).

**Figure 2:**
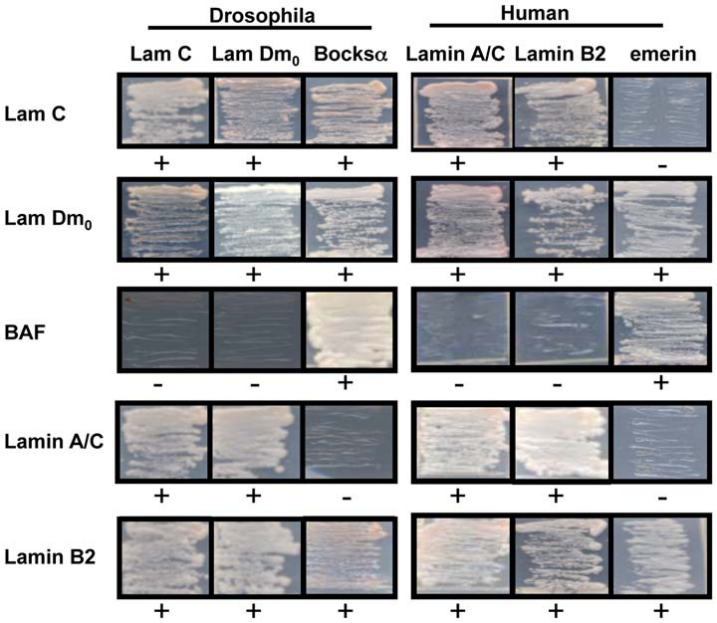
Yeast two-hybrid analysis of nuclear protein interactions. Interactions between human and Drosophila nuclear envelope proteins were tested using yeast two-hybrid analysis. Growth on selective media due to expression of the reporter gene indicates an interaction between the two proteins. + indicates growth; - indicates little to no growth.

The fact that the human proteins exhibited peripheral localization within the Drosophila nucleus suggests conservation of mechanisms of localization. Based on this observation, we predicted that the human and Drosophila lamins would have conserved interaction partners. To test this, yeast two-hybrid analyses were performed. Human Lamin A and C and Lamin B2 each showed interaction with Drosophila Lamin C and Lamin Dm_0_ (Fig. 2), suggesting that the nuclear envelope association could occur through interactions of lamins from the two species. The association between BAF and the LEM domain proteins is also conserved between species, as Drosophila BAF interacts with emerin and Bocksbeutel, a LEM domain protein expressed as two isoforms, one lacking the transmembrane domain [33] (Fig. 2). We did not detect a direct interaction between Drosophila Lamin C with emerin or between human Lamin A/C and emerin in the yeast two-hybrid system, despite reports of their interaction and the mislocalization of emerin in cases of mutated Lamin A/C in cell culture and disease [36]. In contrast, the B-type lamins from both species interacted with emerin and Bocksbeutel (Fig. 2). Thus, many of the interaction partners and mechanisms of localization appear to be conserved between the two species.

### Defects associated with the lack of Drosophila A-type lamin

**Figure 3:**
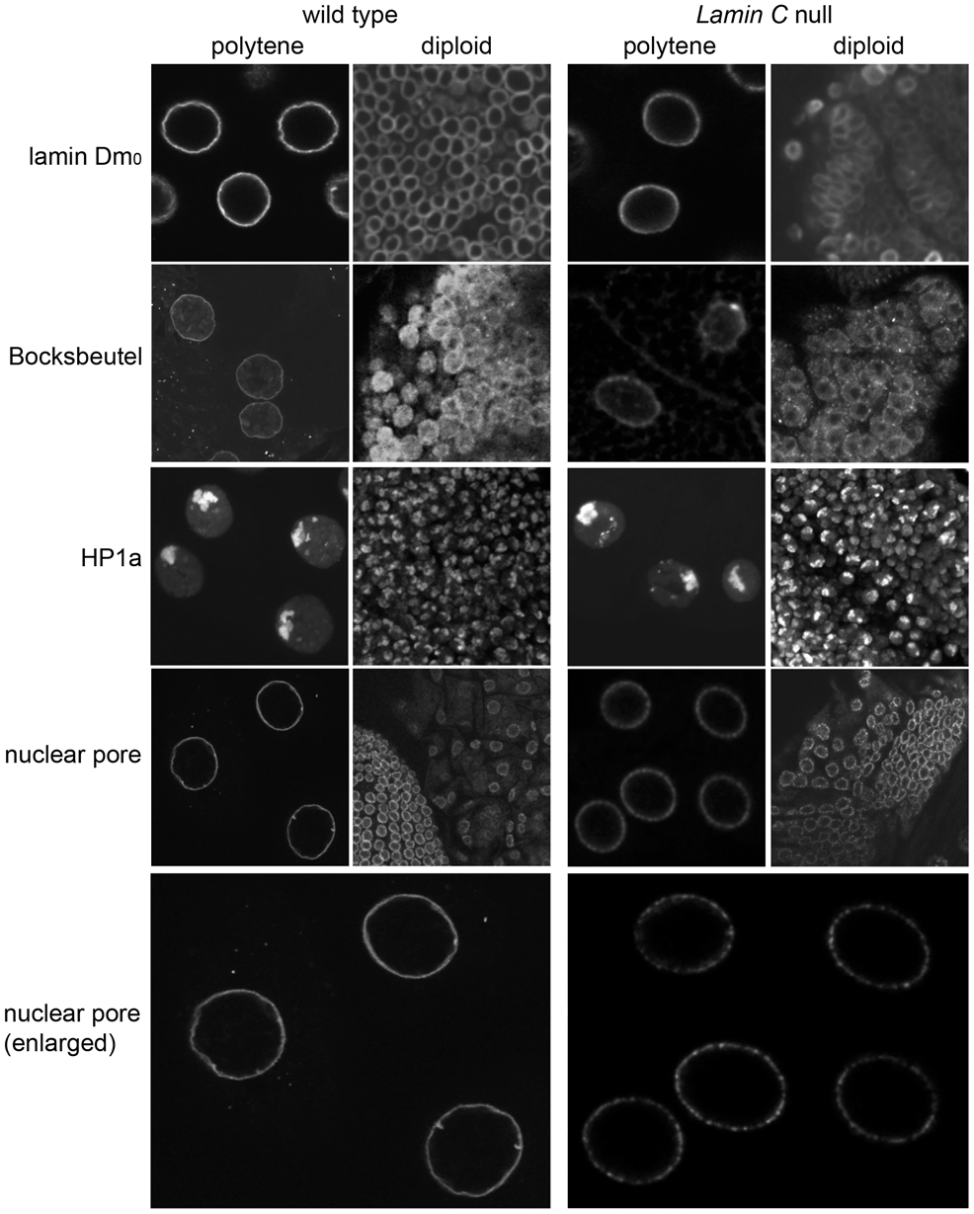
Distribution of nuclear proteins in *Lamin C* nulls. Polytene salivary glands and diploid imaginal disc tissue from wild type larvae and *Lamin C* nulls were stained with antibodies to nuclear envelope associated proteins, lamin Dm_0_, Bocksbeutel and nuclear pores. Heterochromatin organization was determined by staining with antibodies against HP1. An enlarged version of the nuclear pore staining is shown to highlight the peripheral foci that appear in the *Lamin C* null, indicative of nuclear pore clustering.

Mammalian studies have revealed that localization of specific proteins requires intact lamina [10]. To determine whether this reliance upon A-type lamins exists in Drosophila, we tested the localization of chromatin and nuclear envelope proteins in the absence of the Drosophila A-type lamin, Lamin C. Previously we isolated two *Lamin C* null alleles, *Lamin C^EX296^* and *Lamin C^EX187^*, which exhibit a broad lethal phase starting at the second instar and ending at the pre-pupal stage [28]. This relatively late lethal stage allowed us to determine whether the loss of Lamin C altered the localization of other nuclear proteins. Nuclei of Lamin C null larvae were examined by confocal immunofluorescence microscopy following staining with antibodies to various nuclear proteins. Larval imaginal discs and salivary glands, representing diploid and polytene tissue, respectively, were examined. Antibodies against lamin Dm_0_ showed peripheral localization in both polytene and diploid nuclei of wild type and *Lamin C* nulls (Fig. 3), suggesting the two lamin types form independent networks.

In mammalian cells, loss of lamin A/C leads to mis-localization of emerin to the cytoplasm [14]. Therefore, we examined localization of the inner membrane LEM domain protein Bocksbeutel. Peripheral staining was observed in both diploid and polytene nuclei from wild type and *Lamin C* nulls. In Drosophila, loss of the lamin Dm_0_, the B-type lamin, causes nuclear pore clustering [23], therefore, *Lamin C* null larvae were assayed for nuclear pore distribution. Salivary glands from *Lamin C* null larvae showed clustering of the nuclear pores in contrast to the more uniform peripheral staining observed in wild type larvae (Fig. 3). Thus, both A- and B-type Drosophila lamins appear to be responsible for the well-organized distribution of nuclear pores.

Heterochromatin is preferentially located at the nuclear periphery in most eukaryotic cells. In mammalian cells, loss of the gene encoding Lamin A/C causes changes in heterochromatin localization [14]. Antibodies to Heterochromatin Protein 1a (HP1a), a protein enriched within heterochromatin [37], were used to visualize the localization of heterochromatin in wild type larval nuclei and *Lamin C* nulls (Fig. 3). In both cases, a focus of intense staining was observed near the nuclear periphery; this observation is consistent with localization to the chromocenter, the structure formed by the fusion of centromeres in many types of insect cells. Thus, Lamin C does not appear to be essential for peripheral heterochromatin localization in Drosophila.

**Figure 4:**
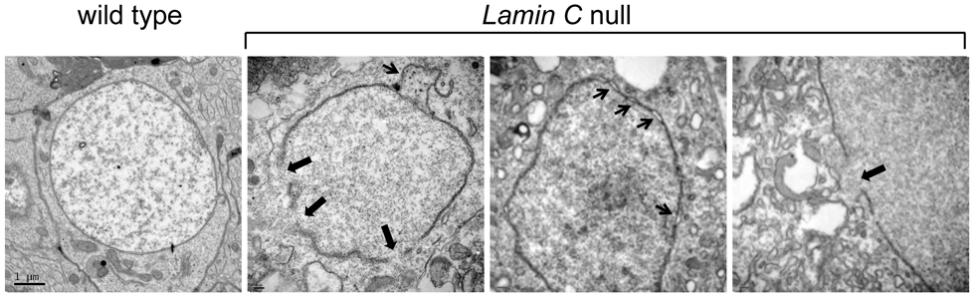
Nuclear defects associated with loss of Lamin C. EM images of nuclei from imaginal discs of second instar larvae. A typical image of a nucleus from a wild type stock (y,*w^67c23^*) surrounded by intact nuclear envelope. Nuclei isolated the *Lamin C* null larvae show separation of the inner and outer membrane (small arrows) and large disruptions in the envelope (large arrows) that allow for chromatin leakage.

To examine the consequences of the loss of Drosophila A-type lamin at higher resolution, electron microscopy (EM) was performed on several tissues of *Lamin C* null larvae. The nuclei of salivary glands and brains of *Lamin C* null larvae showed no differences compared to that of wild type larvae (data not shown). In contrast, abnormalities were observed in the nuclei of imaginal discs (Fig. 4). Fifteen percent of the imaginal disc nuclei showed separation of the inner and outer nuclear membranes and large perforations in the nuclear envelope resulting in “chromatin leakage” (Fig. 4), a phenotype observed in muscle biopsies from EDMD patients [14]. In contrast, minor imperfections in the nuclear envelope were observed in only 2% of nuclei from wild type imaginal discs. The defects observed in imaginal discs are unlikely due to fixation issues, as the endoplasmic reticulum and mitochondrial membranes appeared intact within the samples. It is also unlikely that the nuclear defects represent a general phenotype associated with pending death of the organism since similar analysis of larvae *trans*-heterozygous for mutations in *Su(var)2–5*, the gene encoding HP1a, showed no abnormalities (data not shown). *Su(var)2–5* mutants die at the late third instar larval stage [38], overlapping the lethal phase of *Lamin C* mutants. These data suggest fragility of the nuclear envelope is specific for the lack of Lamin C.

**Figure 5:**
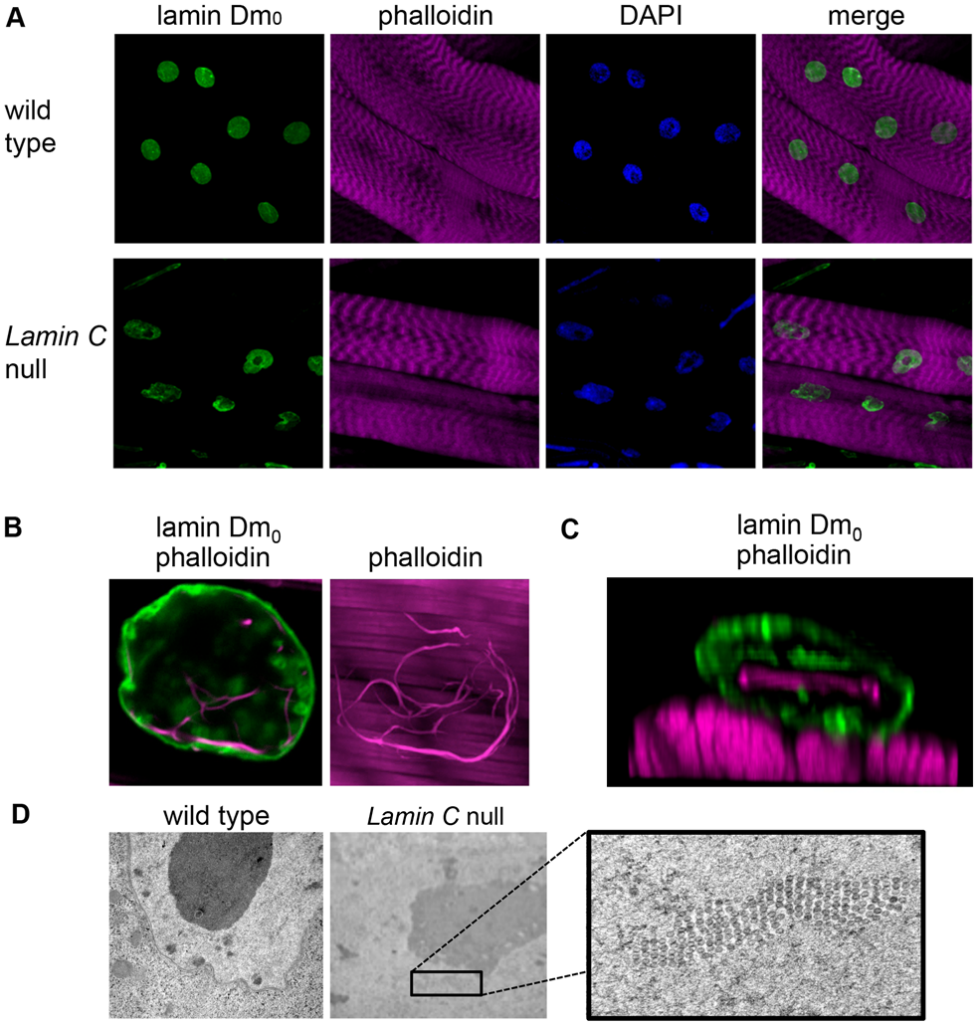
Nuclear and cytoplasmic defects in the larval body wall muscle of *Lamin C* nulls. (A) Confocal images of dissected and fixed larval body wall muscle stained with antibodies to lamin Dm_0_ (green), phalloidin (magenta) and DAPI (blue). (B) Confocal image of a muscle cell nuclei stained with antibodies to Lamin C (green) and phalloidin (magenta). Note the intense filamentous phalloidin staining within the nucleus. Other staining results from actin in the underlying contractile apparatus below the nucleus. (C) Single confocal slice of a muscle nuclei using 3D opacity to visualize lamin Dm_0_ (green) and phalloidin (magenta). Lamin Dm_0_ stains the nuclear periphery; phalloidin stains actin within contractile apparatus below the nucleus and filaments within the nucleus. (D) EM images of nuclei from wild type and *Lamin C* null larval body wall muscle. The boxed insert shows an enlarged cluster of circular particles within the nucleus of *Lamin C* nulls.

Given that several laminopathies are muscular dystrophies, we investigated the structure of larval body wall muscle in *Lamin C* nulls. Confocal microscopy showed abnormal shaped nuclei, as evidenced by irregular anti-lamin Dm_0_ staining in 25% of the muscle nuclei (Fig. 5A). In addition, these nuclei showed phalloidin staining fibers within the nucleus (example shown in Fig. 5B); these fibers were never observed in muscle nuclei of wild type larvae. At the EM level, nuclear envelope ruptures were not apparent as they had been in imaginal disc nuclei. Clusters of ring-like particles were observed within 30% of the nuclei examined (Fig. 5C). In wild type muscle, smaller clusters or single rings were observed in 5% of the nuclei examined. Nearly identical structures have been identified as actin tubules in *Dictyostelium discoideum* spore nuclei [39]. The possibility that the ring-like particles represent actin filaments is consistent with the phalloidin staining observed by confocal microscopy and suggest a possible function for Drosophila A-type lamins in regulating nuclear actin polymerization within the nucleus.

### Modeling laminopathy mutations in Drosophila lamins

**Figure 6:**
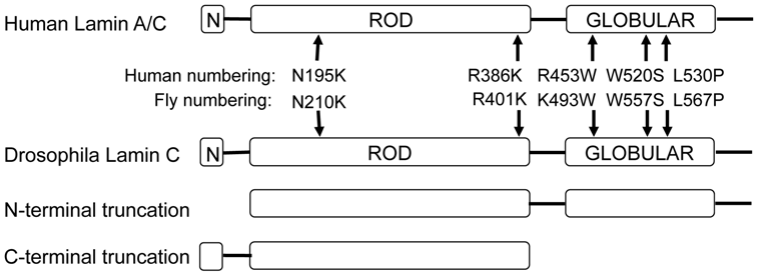
Diagram of mutant forms of Lamin C expressed in transgenic flies. Top shows a diagram of the structure of human LMNA with disease-causing amino acid substitutions indicated. Bottom shows diagrams of the domain structure of Drosophila Lamin C and the mutant forms generated for analyses.

**Figure 7:**
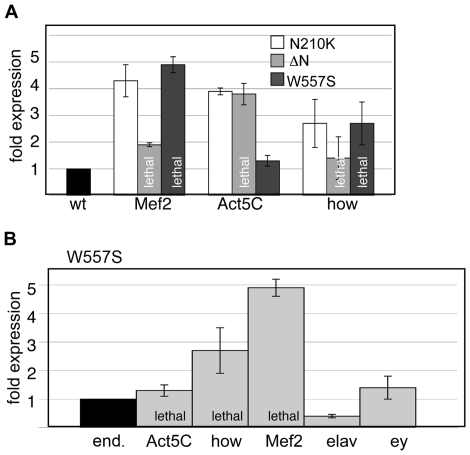
Levels of mutant forms of Lamin C expressed in larvae. (A) Expression of mutant forms of Lamin C under control of three different Gal4-UAS drivers. The top shows a representative western of protein extract from third instar larvae containing the Mef2 or Act5 driver in combination with a transgene encoding NI95K, W520S or the N-terminal truncation (ΔN). Extract from the y,w67c23 host injection stock is used for comparison of endogenous Lamin C (end.). The bottom shows a graphical representation of quantitative analysis of westerns performed on three independently generated protein extracts for each genotype. The average value is plotted with error bars representing standard error of the mean. These results demonstrate that lethality caused by the N-terminal deletion and W557S is not a general result of over-expression. (B) Expression of W557S under control of five different drivers. The top representative western analysis of protein extracts from third instar larvae expressing a transgene encoding the W557S mutant form of Lamin C under the control of various Gal4 drivers. The bottom represents graphical representation of quantitative analysis of westerns performed on three independently generated protein extracts from larvae possessing the W557S expressing transgene and various drivers. The average value is plotted with standard error of the mean. These results indicate that the semi-lethal phenotype associated with W557S when expressed ubiquitiously or specifically in muscle does not correlate with levels of expression.

**Figure 8:**
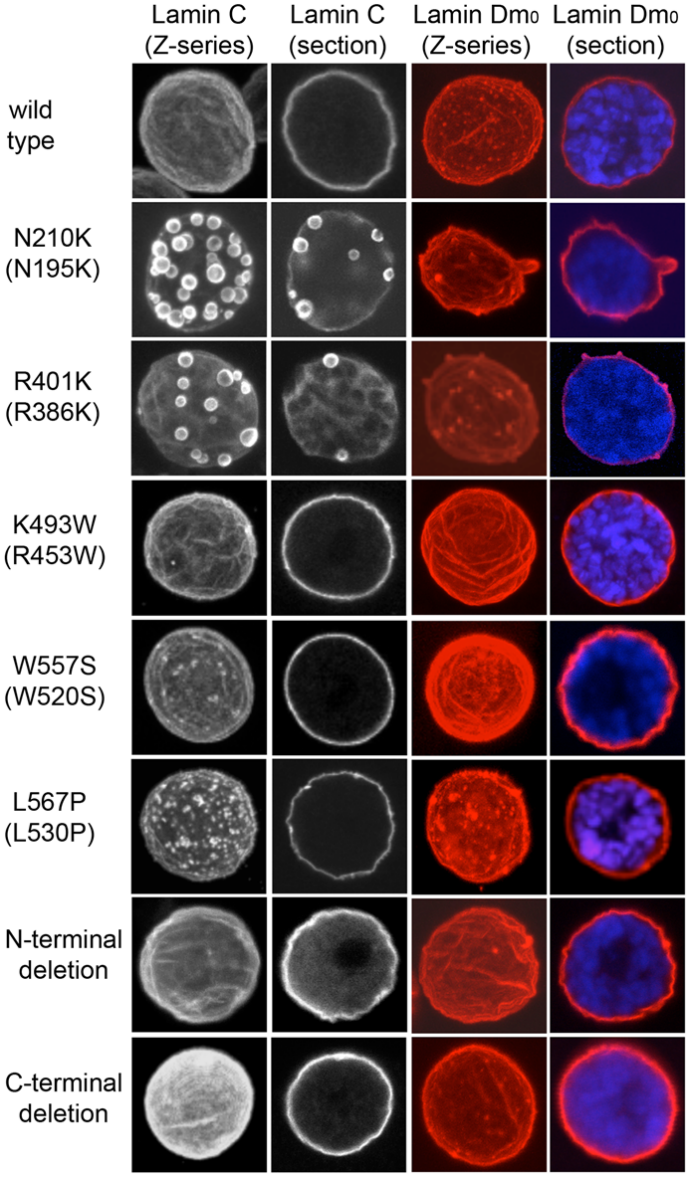
Nuclear localization of the mutant forms of Lamin C and lamin Dm_0_. Salivary gland nuclei from larvae expressing wild type or mutant forms of Lamin C under control of a *hsp70* promoter were fixed and stained with antibodies to Lamin C (white), lamin Dm_0_ (red) and TOPRO that detects DNA (blue).

One approach to modeling human disease conditions in Drosophila is to express mutant versions of the human protein responsible for disease in transgenic flies. Since our studies indicated both conserved and possible species-specific functions for human and Drosophila lamins, we opted to model the human mutations in the Drosophila lamin proteins. Seventy of the Lamin A/C amino acid residues altered in disease human disease are conserved in Drosophila Lamin C. Therefore, we modeled the human disease-causing mutations in Lamin C by making the corresponding amino acid substitutions in the Drosophila proteins. We modeled amino acid substitutions within the rod and C-terminal tail that give rise to AD-EDMD (R386K, R453W, W520S and L530P, human numbering). In addition, we generated an amino acid substitution within the rod domain that causes DCM (N195K, human numbering) and two truncations that remove either the N-terminal head or the C-terminal globular domain (Fig. 6). Loss of the N-terminal head domain has been associated with a neurogenic variant of AD-EDMD [40] as well as defects in DNA replication and transcription [41], [42]. Loss of the C-terminal domain allowed us to assess the function of the Ig-fold [43]. Transgenes encoding these mutant forms of Lamin C were expressed under control of the *hsp70* promoter or the Gal4-UAS system [35] (Figs. 7, 8 and Fig. S1).

We determined whether expression of mutant forms of Lamin C altered nuclear morphology. Third instar larvae expressing these mutant proteins under control of the *hsp70* promoter were heat shocked and salivary glands were stained with antibodies to Drosophila Lamin C and lamin Dm_0_. Wild type Lamin C showed the anticipated localization to the nuclear periphery and nucleoplasm (Fig. 8). N210K (human N195K) and R401K (human R386K) showed nuclear aggregates and reduced staining in the nuclear periphery. The presence of Lamin C aggregates correlated with aggregation of lamin Dm_0_ (Fig. 8), consistent with earlier studies [28]. In contrast, K493W (human R453W), W557S (human W520S), and both the N- and C-terminal truncations showed accumulation at the nuclear periphery. L567P (human L530P) accumulated at the periphery, but also formed speckles that were dissimilar to the large aggregates observed with amino acid substitutions within the rod domain (Fig. 8). Similar speckles were observed upon expression of L530P in mammalian cells [44]. Based on these results, we conclude that amino acid substitutions within the rod domain give rise to nuclear aggregates that are likely the result of aberrant lamina assembly. Interestingly, the formation of the nuclear aggregates did not correlate with lethality. In fact, lethal phenotypes were only observed with mutant lamins that showed typical peripheral localization.

One explanation for the tissue-restricted phenotypes associated with laminopathies is that mutant lamins disrupt tissue-specific functions. To determine whether mutant forms of Drosophila A-type lamin cause tissue-specific defects, we used the Gal4-UAS expression system [35] . Effects of ubiquitous expression early in development were tested using the Actin 5C (Act5C) driver. Tissue-specific expression was tested using the larval imaginal disc driver T80 [45], the muscle-specific drivers Myocyte enhancing factor 2 (Mef2) and held out wings (how^24B^) and the eye-specific driver eyeless (ey) (Flybase). Given that mutations in human *LMNA* also produce neuropathies we tested for central nervous system effects using the pan-neural driver elav [46], [47]. Multiple transgenic stocks expressing wild type and mutant forms Lamin C were tested to rule out effects due to the site of insertion (Table S2). The levels of wild type or mutant protein produced from a given driver were nearly equivalent (Fig. 7 and Fig. S1).

Expression of the N-terminal truncation and W557S (human W520S) using the Act5C and T80 drivers resulted in lethality or semi-lethality (Table 1). Flies expressing all other mutant proteins or the wild type control were viable (Table 1). Lethality was also observed for the N-terminal truncation and W557S with the muscle-specific drivers Mef2 and how^24B^, suggesting that the lethality associated with ubiquitous expression was likely due to expression in muscle. In contrast, no lethality or visible phenotypes were observed using elav or ey drivers that are expressed in the nervous system and eye, respectively. Lethality could not be attributed to differences in protein levels (Fig. 7). For example, expression of W557S by the Act5C driver caused lethality, yet Lamin C protein levels were nearly equal to that of endogenous Lamin C and to that produced by the ey driver, which allowed for viability (Fig. 7). In addition, the relative levels of N210K and the N terminal truncation produced by the Act5 driver were nearly equivalent, yet only the N-terminal truncation caused lethality (Fig. 7). Collectively, these data strongly suggest an essential role of the N-terminal globular domain and the Ig-fold in muscle.

**Table 1:** Results of tissue-specific expression of mutant forms of Lamin C.

	Act 5C	T80	How^24B^	Mef2	elav	ey
wt	viable	viable	viable	viable	viable	viable
N210K (N195K) rod	viable	viable	viable	viable	viable	viable
R401K (R386K) rod	viable	viable	viable	viable	viable	viable
K493W (R453W) globular	viable	viable	viable	viable	viable	viable
W557S (W520S) globular	semi-lethal*	semi-lethal	semi-lethal	lethal	viable	viable
L567P (L530P) globular	viable	viable	viable	viable	viable	viable
N-terminal truncation	lethal	lethal	lethal	lethal	viable	viable
C-terminal tranucation	viable	viable	viable	viable	viable	viable

* Semi-lethal denotes 5 to 60% viability of the expected class based on Mendelian ratios.

## Discussion

### Human nuclear envelope proteins incorporate normally into the insect nuclear envelope

Mutations in A-type lamins lead to disease in humans. Given the structural similarities between A-type lamins in humans and flies, and the fact that, like humans, Drosophila is the only invertebrate known to possess two lamin types it was of interest to determine whether human lamins would properly localize in an insect nucleus. Expression of the human proteins Lamin A, Lamin C, Lamin B2 and emerin in transgenic Drosophila exhibited a pattern of localization similar to that observed in humans. In addition, yeast-two hybrid data showed that many of the interactions between lamins and nuclear envelope associated proteins are conserved (Fig. 2).

One difference between the human and Drosophila A-type lamins involves protein processing. Human Lamin C is produced without a CaaX box due to alternative splicing, and is therefore never prenylated for membrane anchorage. Human lamin A is initially produced with a CaaX box (designated pre-lamin A) that is modified and subsequently cleaved to produce mature lamin A lacking a CaaX box [3]. In contrast, Drosophila Lamin C lacks a CaaX box, while Drosophila lamin Dm_0_ possesses a CaaX box. Due to conservation of prenylated consensus sequences, it is likely that the human lamin A is prenylated in Drosophila, however, the occurrence of downstream cleavage events is not yet known. The localization of human Lamin A in Drosophila most closely resembles that of Drosophila lamin Dm_0_, suggesting that while the CaaX is processed for anchorage, it is not subsequently cleaved as it appears to be in human cells. The localization of human Lamin C, lacking a CaaX box, displays internal nuclear localization, which is similar to that observed for Drosophila Lamin C. Lamin B2 shows somewhat aberrant localization, suggesting difficulties in assembly, which nevertheless, under controlled heat shock treatment did not affect viability. It is interesting to note, however, that human B-type lamins, when over-expressed by ubiquitous or muscle-specific Gal4 drivers, cause lethality, whereas over-expression of A-type lamins and emerin using the same drivers has no effect (Table S1).

### Loss of Lamin C causes nuclear defects

Several analyses have been performed on Drosophila *lamin Dm_0_* mutants [24]. In contrast, limited characterization had been performed on *Lamin C* nulls. Given that Drosophila is being developed as a model for human disease [48] and recently a focus for laminopathies [28], [48], [49], [50], we wanted to better characterize the Drosophila *Lamin C* null phenotype with respect to nuclear morphology. Ultrastructural analyses of Drosophila nuclear envelopes lacking Lamin C revealed defects in the nuclear envelope and the nuclear interior. Nuclear envelope defects were observed in diploid imaginal disc tissue. These defects included separation of the inner and outer nuclear envelope and membrane disruptions allowing for chromatin leakage (Fig. 4). These phenotypes are consistent with those observed in *Lmna−/−* mouse knock-out cells and muscle tissue from laminopathy patients [14], [51], [52], [53]. The phenotypes observed in flies are restricted to specific cell types; nuclear envelope detachment phenotypes were observed in imaginal disc nuclei where Lamin C is expressed, but not in nuclei of brains that normally express low levels of Lamin C [30]. Separation of the inner and outer nuclear membrane might result from loss of connections between the inner and outer membrane. A LINC (linker of nucleoskeleton and cytoskeleton) complex has recently been identified that connects the cytoplasm to the nucleoplasm through a complex of bridging proteins [29], [47], [54]. This complex consists of SUN domain proteins that span the inner nuclear envelope and interact with A-type lamins on the inner side of the nuclear envelope [55]. Within the perinuclear space, SUN domain proteins interact with nesprins. Nesprins span the outer nuclear envelope and make contacts with the actin cytoskeleton network. Drosophila possesses both SUN and nesprin proteins, providing the possibility for similar interactions [26], [56], [57], [58]. The expansion of the perinuclear space observed in the *Lamin C* null (Fig. 2) is similar to that observed upon RNAi knock-down of the inner nuclear envelope proteins Sun1 and Sun2 in mammalian cells [54]. Loss of A-type lamin is predicted to cause mis-localization of the Sun domain proteins and/or destabilization of the Sun-nesprin interactions, allowing for expansion of the perinuclear space. This idea is supported by the mislocalization of Sun2 in *Lmna−/−* MEF cells [54], [59].

The existence of a connection between the nucleus and cytoplasm is consistent with the fact that *Lamin C* nulls show muscle cell nuclear envelope defects (Figs. 4 and 5). In these mutants, clustered ring-like particles within the nuclei of the larval body wall muscles are observed (Fig. 5C) are remarkably similar to those observed in several types of Drosophila cells, however, the biochemical composition of these particles was not determined [60], [61], [62]. Nearly identical structures were discovered in Dictyostelium spores and determined to be tubular actin structures [39]. The phalloidin staining fibers observed in Lamin C null larval muscle nuclei are consistent with the presence of filamentous actin in the nucleus. Taken together, we hypothesize that A-type lamins might regulate the import/export and or polymeraization of actin within the nucleus. Consistent with this hypothesis, A-type lamins have been reported to interact with actin [10].

### Expressing laminopathic mutant lamins in Drosophila

The conserved properties of A-type lamins in Drosophila and humans support an analysis of laminopathic mutants in a Drosophila model, to shed light on general underlying properties of the nuclear envelope that relate to disease. Specific amino acid substitutions were selected due to their location within a given domain of the protein and their connection with human disease. Expression of Drosophila Lamin C possessing amino acid substitutions within the rod domain caused lamin aggregation. This aggregation is strikingly similar to that observed upon expression of human A-type lamin rod domain mutants in mammalian cell culture and is consistent with earlier findings [28], [63]. Despite the presence of Drosophila Lamin C aggregates, flies expressing rod domain amino acid substitutions were viable (Table 1) and showed no visible adult phenotypes. In contrast, lethality was observed upon expression of the N-terminal truncation and W557S amino acid substitution within the C-terminal globular domain. The fact that these forms cause lethality in the background of wild type endogenous Lamin C suggests they are functioning in a dominant negative manner. Consistent with our finding, an N-terminal truncation of human lamin A/C has dominant negative effects in human cell culture and *in vitro* lamin assembly systems [41], [64]. In addition, the first 20 amino acids of the mouse Lamin A head domain were shown to be critical for lamin organization [65] . In humans W520S causes AD-EDMD [66]. This amino acid substitution resides within the Ig-fold, a domain that is likely to play a role in lamin assembly and to bind DNA [7], [43], [67]. Based on the structure of the Ig-fold [7], this residue resides within a groove. Lethality is observed with W557S, but not the C-terminal truncation, suggesting lethality is not due to loss of domain function. The fact that expression of W557S causes lethality when expressed in muscle, but not in non-muscle tissues (Table 1), implies a role for this residue in muscle development, possibly by generating a structure needed for muscle-specific protein interaction. Collectively, these transgenic Drosophila studies have identified the N-terminal head and the Ig-fold domain as playing roles in muscle function.

Our studies and those of others suggest that Drosophila will be useful for modeling laminopathies [22], [49]. Here, we focused on Lamin C and discovered many commonalities between the fly and human A-type lamins. The loss of A-type lamins in both species gives rise to nuclear envelope fragility. Expression of mutant forms in both humans and flies give rise to similar nuclear abnormalities and muscle defects. We also find differences between the two species. For example, alterations in A-type lamins in humans correlates with changes in heterochromatin organization [68], whereas, Drosophila nuclei lacking Lamin C showed no obvious changes in heterochromatin (Fig. 4 and 5). Amino acid substitutions in the rod domain cause muscle defects in humans, whereas, flies expression theses amino acid substitutions appear normal. Whether such differences can be attributed to species-specific functions remains to be discerned. Certainly, for physiological aspects of EDMD, Drosophila presents as a promising model.

## Materials and Methods

### Genetic Analyses and transgenic stocks

Drosophila stocks were raised on standard sucrose/cornmeal medium. Generation of transgenic stocks and heat shock induced expression were as previously reported [28]. For transgene studies, mutant forms of Drosophila Lamin C were generated via Quikchange site directed mutagenesis (Stratagene) using *Lamin C* as a template. Except for *LMNB2*, the genes encoding human lamins and emerin were amplified using Pfu-Ultra (Stratagene) from human cDNA libraries. A full-length cDNA encoding Lamin B2 was purchased from a commercial source (AATC). All Drosophila and human cDNAs were cloned into pUAST and pCaSpeR-hs-act transformation vectors. The N-terminal truncation used here differed from that previously published [28]. The previous version was modeled after constructs used in mammalian systems that deleted the N-terminal domain and eight amino acids of the rod domain [64]. Aggregation of Lamin C observed upon expression of this mutant was likely due to removal of the amino acids within the rod domain, as amino acid substitutions within the rod show a nearly identical phenotype. The construct here produces a Lamin C protein lacking only the first 42 amino acids that represent only the globular head domain. Expression of this mutant form does not shown nuclear aggregates. The C-terminal deletion removes the last 194 amino acids. The designations for all stocks used here are shown in Table S2.

### Yeast-two-hybrid analyses

Full-length cDNAs encoding nuclear envelope proteins were cloned into the yeast two-hybrid bait and/or prey vectors pGBKT7 (BD) and pGADT7 (AD) (MATCHMAKER GAL4 Two-Hybrid System 3, CLONTECH). AH109 yeast cells were transformed with bait, grown in appropriate media, made competent and then co-transformed with prey following the protocol from ZYMO Research (Frozen-EZ Yeast Transformation II^TM^). The transformation mixture was plated on low stringency plates (-Leu/-Trp) plates and incubated at 30° C for 2–4 days. Colonies were selected and streaked on high stringency (-Ade/-His/-Leu/-Trp) plates to score for interactions. Plates were incubated at 30° C for up to 5 days and then scored for growth.

### Tissue preparation and microscopy

Confocal images were obtained from dissected and immunostained salivary glands, imaginal discs and brains from larvae. Tissues were dissected in cold 1X PBS and transferred to 2% paraformaldehyde for 10–20 minutes. After several washes in 1X PBS, the tissues were blocked for a minimum of 60 minutes in 1% BSA in 1X PBS at room temperature. Tissues were then incubated in primary antibody either overnight at 4°C or at room temperature for at least an hour. After several washes in 1X PBS, the tissues were blocked in 1% BSA and then incubated with a fluorescent secondary antibody for two to four hours in a light tight box at room temperature. Primary and secondary antibody dilutions contained 1%BSA in 1XPBS. After a final wash, tissues were transferred to slides and mounted in Vectashield (Vector Labs) under shimmed coverslips. Antibodies and dilutions were as follows: Drosophila Lamin C: LC28.26 at 1∶400. Drosophila lamin Dm_0_ ADL84.12 or ADL67.10 used at 1∶400; Drosophila HP1 (C1A9) at 1∶100; Drosophila Bocksbeutel gIII at 1∶1000. All Drosophila antibodies except Bocksbeutel were raised in mouse, and obtained from the University of Iowa Hybridoma Core facility. The Bocksbeutel antibody was raised in guinea-pig, and was a generous gift from Nicole Wagner and Georg Krohne (University of Wurzburg) [33]. Nuclear pore proteins were detected using MAb414 (anti-mouse, Covance) at a dilution of 1∶3000. For analysis of human proteins, the following primary antibodies were used: Lamin A and C: N-18; sc-6215 (donkey anti-goat, Santa Cruz) used at 1∶3000; Lamin B1 and B2: NA12 (anti-mouse, Oncogene Research) at 1∶3000. This antibody is reportedly specific for Lamin B1, but did not detect a protein in our extracts from flies possessing a LMNB1 transgene (data not shown). In addition, this antibody weakly detected Lamin B2 in transgenic stocks. Secondary antibodies included Rhodamine Red and Cy3 (Jackson Immunoresearch) at a dilution of 1∶1000; AlexaFluors (546, 596 and 647) and TOPRO (Molecular Probe) at dilutions of 1∶1000 or higher. DAPI staining was performed at 250 µg/µl for approximately 30 seconds. Images were collecting on a Bio-Rad MRC 1024 confocal lasar scanning imaging system (Center for Microscopy, University of Iowa) using a 63X objective. Confocal images are represented as the average projection of a stack of sections (Z-series) or as a single section.

Larval body wall muscle was prepared according to published procedures [69]. Fixed muscle was stained with antibodies to Lamin Dm_0_ (ADL84.12, 1∶500 dilution), Lamin C (LC28.26, 1∶500 dilution) and Texas Red-X phalloidin (1.67 µM, Invitrogen). Secondary antibodies were Alexa Fluor 488-conjugated goat anti-mouse Ig-G (1∶400, Invitrogen) and Alexa Fluor 546-conjugated donkey anti-rabbit Ig-G (1∶400, Invitrogen). Imaging was performed on a Bio-Rad MRC 1024 confocal microscope.

For transmission electron microscopy (EM) of tissues, wild type and *Lamin C^EX187^* larvae were collected at the second instar stage. *Lamin C^EX187^* possesses a deletion within the first exon and is a protein null [28]. Approximately 100 to 150 imaginal discs or brains were placed in one ml of fixation solution (2% gluteraldehyde, 0.1 M Na cacodylate, ph 7.2) and left at 4°C overnight. Subsequent tissue preparations were performed by the University of Iowa Center for Microscopy. Muscle fiber EM was performed by fixing third instar larval body wall preparations in Trumps fixative (4% paraformaldehyde/2% gluteraldehyde in cacodylate buffer) for 2.5 hours. Samples were then osmicated in 1% osmium in 0.1M cacodylate buffer for 30 minutes, dehydrated, embedded in Spurs resin and thin sectioned at ∼80 nm.

### Western analyses

To determine the levels of Lamin C, protein extracts from larvae were prepared [70] and analyzed with antibodies to Lamin C (LC28.26 anti-mouse IgG) [21] used at 1∶5000 dilution. Antibodies to alpha-tubulin (anti-mouse IgG1, T5168, Sigma) were used at 1∶400,000 dilution as a control for protein loading. An HRP-conjugated anti-mouse IgG (cat. #31446, Pierce) used at 1∶20,000 dilution served as a secondary antibody. Detection was carried out using the SuperSignal West Pico chemiluminescent substrate (cat. #34080, Pierce). Signals on the westerns were imaged using an Epi Chemi II darkroom unit fitted with a CCD camera (UVP) and quantitated using LabWorks Image Acquisition software (UVP). At least three independent protein isolations were performed for each genotype. Lamin C expression was normalized against that of alpha-tubulin. The percent relative expression was calculated by dividing each normalized value by the normalized value of wild type, which was set at 100%.

## Supporting Information

Table S1 (0.03 MB DOC)

Table S2 (0.06 MB DOC)

Figure S1 Levels of wild type and mutant forms of Lamin C expressed from the Mef2 larval muscle-specific driver. (A) Representative western analysis of protein extract from third instar larvae containing the Mef2 driver in combination with a transgene encoding wild type (WT) Lamin C, W520S, N195K or Lamin C ΔN. Extract from y,w67c23 host injection stock was used for comparison of endogenous levels of Lamin C (end.). Larvae expressing full length Lamin C possess a truncated break-down product that is similar in size to Lamin C ΔN (B) Graphical representation of quantitative analyses of westerns performed on three independently generated protein extracts for each genotype. The average value is plotted with error bars representing standard error of the mean.
(1.99 MB TIF)
