# Supplementary material for: A Comparative Study of Drosophila and Human A-Type Lamins
Source: PLoS One. 2009 Oct 26;4(10):e7564. doi: 10.1371/journal.pone.0007564 (PMC2762312; doi:10.1371/journal.pone.0007564)
Supplement: Table S1 — (0.03 MB DOC) [file pone.0007564.s001.doc]

Supplemental Table 1. Results of GAL4 driven expression of Drosophila expressing human lamin proteins

| Human transgene | Driver | | |
| --- | --- | --- | --- |
|  | ACT5C | MEF2 | ELAV |
| LMNA 10R-55^1A^ | Viable | Viable | Viable |
| LMNA 10R-55^9^ | Semi-lethal  (56% escapers) | Viable | Viable |
| LMNC 11-111^5C^ | Viable | Viable | Viable |
| LMNC 11-111^2^ | Viable | Viable | Viable |
| LMNB2 18-11^6^ | Semi-lethal  (8% escapers) | Lethal | Viable |
| LMNB2 18-20^3^ | Lethal | Lethal | Viable |
| Emerin 40-83M1 | Viable | Viable | ND |
| Emerin 40-6F2 | Viable | Viable | ND |

*Semi-lethal denotes 8 to 60% viability of the expected class based on Mendelian ratios. ND, not determined
