# Supplementary material for: A Comparative Study of Drosophila and Human A-Type Lamins
Source: PLoS One. 2009 Oct 26;4(10):e7564. doi: 10.1371/journal.pone.0007564 (PMC2762312; doi:10.1371/journal.pone.0007564)
Supplement: Table S2 — (0.06 MB DOC) [file pone.0007564.s002.doc]

Supplemental Table 2. Summary of transgenic stocks.

| Transgene | Vector | Location | Vector | Location |
| --- | --- | --- | --- | --- |
| Drosophila (human no.) | pCaSpER-hs-ACT | Chromosome | pUAST | Chromosome |
| Wild type | HS-LamC.6 | X | LamC.3 | 3 |
|  | HS-LamC.22 | X | LamC.9 | 2 |
| N210K (N195K) | N195K.20 | X | N195K.6 | 3 |
|  | N195K.29 | 2 or 3 | N195K.12 | X |
| R401K (R386K) | R386K.44 | X | R386K.16 | 3 |
|  | R386K.49 | 3 |  |  |
| K493W (R453W) | 2-60^5^ | X | 14-87^7^ | X |
|  | 2-2^1A^ | 3 | 14-34^1^ | 3 |
| W557S (W520S) | 3-28^1^ | 3 | 16-45^4^ | 2 |
|  | 3-47^1D^ | ? | 16-98^2^ | X |
| L567P (L530P) | 4-7^11^ | X | 15-90^8^ | X |
|  | 4-7^4^ | 2 | 15-90^4^ | 3 |
| N-term truncation | 31-27F | 3 (balanced) | 37-11F1 | X |
|  | 31-40F | 3 | 37-23M1 | 2 |
| C-term truncation | 27-1^3^ | X | 28-4^3^ | X |
|  |  |  | 28-4^6^ | 3 (two inserts) |
| Human LMNA | 8-27^2^ | X | 10R-55^1A^ | X |
|  | 8-52^1^ | X | 10R-55^9^ | 3 |
| Human LMNC | 7-11^1^ | 3 | 11-111^2^ | X |
|  | 7-16^1^ | 2 | 11-111^5C^ | 3 |
| Human LMNB1 | 6-20^3^ | 3 | 9-45^2A^ | 3 (two inserts) |
|  | 6-39^13^ | 3 |  |  |
| Human LMNB2 | 17-27^5CB^ | 3 (balanced) | 18-11^6^ | 3 |
|  | 17-27^1^ | X | 18-20^3^ | 3 |
| Human emerin | 34-29M1 | X | 40-6F4 | X |
|  | 34-57M1 | 3 | 40-83M1 | 3 |
