# Supplementary figures and images for: A Comparative Study of Drosophila and Human A-Type Lamins
Source: PLoS One. 2009 Oct 26;4(10):e7564. doi: 10.1371/journal.pone.0007564 (PMC2762312; doi:10.1371/journal.pone.0007564)

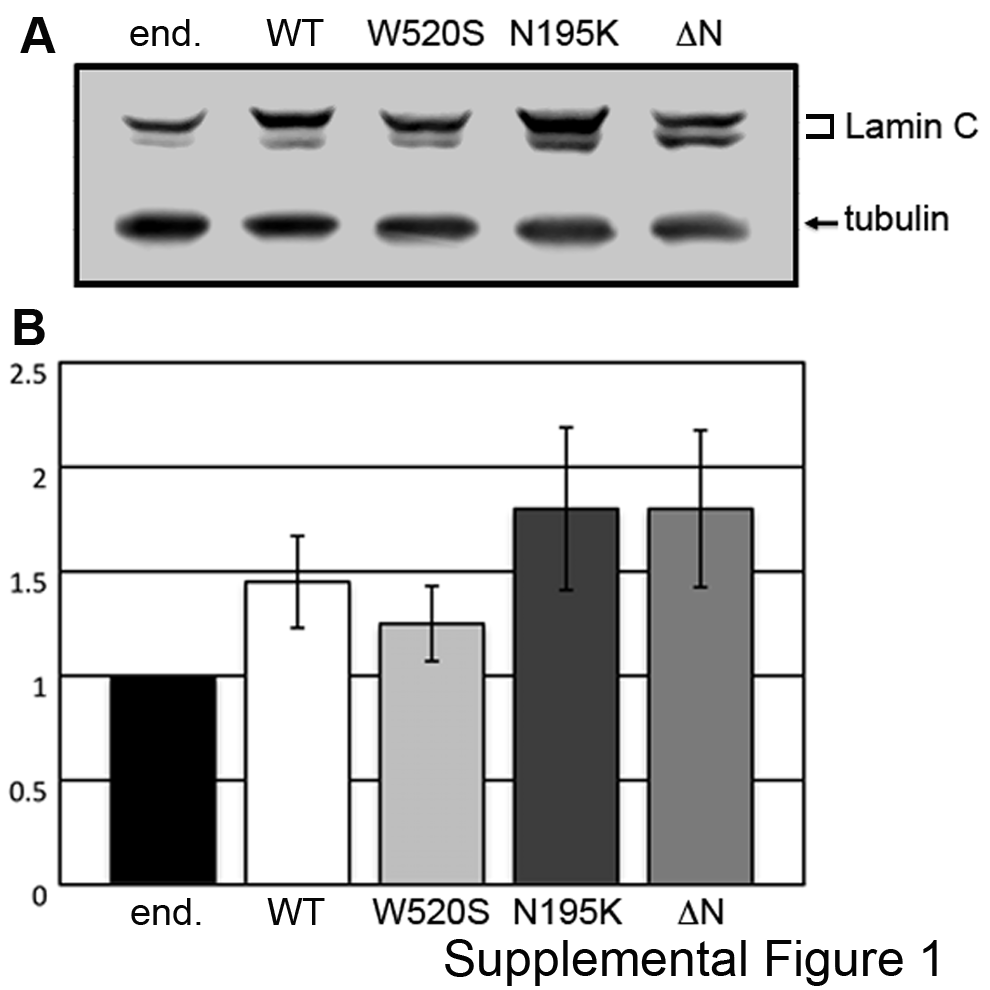

Supplement: Figure S1 — Levels of wild type and mutant forms of Lamin C expressed from the Mef2 larval muscle-specific driver. (A) Representative western analysis of protein extract from third instar larvae containing the Mef2 driver in combination with a transgene encoding wild type (WT) Lamin C, W520S, N195K or Lamin C ΔN. Extract from y,w67c23 host injection stock was used for comparison of endogenous levels of Lamin C (end.). Larvae expressing full length Lamin C possess a truncated break-down product that is similar in size to Lamin C ΔN (B) Graphical representation of quantitative analyses of westerns performed on three independently generated protein extracts for each genotype. The average value is plotted with error bars representing standard error of the mean. (1.99 MB TIF) [file pone.0007564.s003.tif]
